# Sequence Analysis of pKF3-70 in *Klebsiella pneumoniae*: Probable Origin from R100-Like Plasmid of *Escherichia coli*

**DOI:** 10.1371/journal.pone.0008601

**Published:** 2010-01-06

**Authors:** Huiguang Yi, Yali Xi, Jing Liu, Junrong Wang, Jinyu Wu, Teng Xu, Wei Chen, Biaobang Chen, Meili Lin, Huan Wang, Mingming Zhou, Jinsong Li, Zuyuan Xu, Shouguang Jin, Qiyu Bao

**Affiliations:** 1 Institute of Biomedical Informatics/Zhejiang Provincial Key Laboratory of Medical Genetics, Wenzhou Medical College, Wenzhou, China; 2 T-Life Research Center, Fudan University, Shanghai, China; 3 Department of Molecular Genetics and Microbiology, University of Florida, Gainesville, Florida, United States of America; Singapore Immunology Network, Singapore

## Abstract

**Background:**

*Klebsiella pneumoniae* is a clinically significant species of bacterium which causes a variety of diseases. Clinical treatment of this bacterial infection is greatly hindered by the emergence of multidrug-resistant strains. The resistance is largely due to the acquisition of plasmids carrying drug-resistant as well as pathogenic genes, and its conjugal transfer facilitates the spread of resistant phenotypes.

**Methodology/Principal Findings:**

The 70,057 bp plasmid pKF3-70, commonly found in *Klebsiella pneumoniae*, is composed of five main functional modules, including regions involved in replication, partition, conjugation, transfer leading, and variable regions. This plasmid is more similar to several *Escherichia coli* plasmids than any previously reported *K. pneumoniae* plasmids and pKF3-70 like plasmids share a common and conserved backbone sequence. The replication system of the pKF3-70 is 100% identical to that of RepFII plasmid R100 from *E. coli*. A beta-lactamase gene *ctx-m-14* with its surrounding insertion elements (ISEcp1, truncated IS903 and a 20 bp inverted repeat sequence) may compose an active transposon which is directly bordered by two putative target repeats “ATTAC.”

**Conclusions/Significance:**

The *K. pneumoniae* plasmid pKF3-70 carries an extended-spectrum beta-lactamase gene, *ctx-m-14*. The conjugative characteristic makes it a widespread plasmid among genetically relevant genera which poses significant threat to public health.

## Introduction

*Klebsiella pneumoniae*, the second most clinically significant pathogen after *Escherichia coli*, is also considered to be the most important iatrogenic pathogen and its infection could serve as an indication of hospital-acquired infection [1]. In recent years, there has been an increased tendency for *K. pneumoniae* infection in neonatal wards, resulting in septicemia and meningitis which cause serious public health threats [2]. Moreover, as a result of widespread and large-dose usage of various antibiotics in clinical settings, multidrug-resistant strains are emerging, increasing the difficulty of treating such bacterial infections [3]. Recently, extended-spectrum beta-lactamase (ESBL) genes have been more and more frequently identified in *K. pneumonia* strains as well as other bacteria [4]. As ESBL enable its host to resist a broad spectrum of antibiotics, the ESBL-carrying *K. pneumonia* strains are highly dangerous, especially among long term care patients where it could cause an iatrogenic infection outbreak. Genes for antibiotic resistance and pathogenicity are usually plasmid-borne. Thus it will be of a further threat to public health when such plasmids are conjugative [5], as it allows pathogenicity and resistance genes to disseminate across strains, species and even genera [6], [7].

Recently, we have collected 206 multidrug-resistant *K. pneumoniae* strains from Wenzhou China, covering the period of year 2002 to 2006, and subjected to analysis for their drug resistance spectra and plasmid profiles. Most of those harbored three plasmids, large (140 kb), medium (90 kb) and small (70 kb) (data not shown). One multidrug-resistant strain, named *K. pneumoniae* KF3, was chosen as a representative and its three plasmids were sequenced. In this report, we analyzed its smallest plasmid pKF3-70. It consists of five main functional modules, including replication, partition, conjugation, transfer leading and variable regions. pKF3-70 is distantly related to other *K. pneumoniae* plasmids but closely related to some *E. coli* plasmids and the pKF3-70 like plasmids share common backbone structure. Our results indicate that pKF3-70 is a conjugative plasmid which could transfer to bacteria of different genera and disseminates clinically significant genes.

## Results/Discussion

### General Features

Plasmid pKF3-70 is a 70,057 bp circular plasmid with an average GC content of 52.3%, and there are regions with atypical higher or lower strength of GC content (Figure S1, Table S1). It encodes 95 putative open reading frames (ORFs) which can be divided into five functional regions: partition region (ORF1, 2), replication region (ORF15, 16), conjugation region (ORF21-60), transfer leading region (ORF61-95) and variable region (ORF4-14, Table S1).

### Replication and Partition

**Figure 1:**
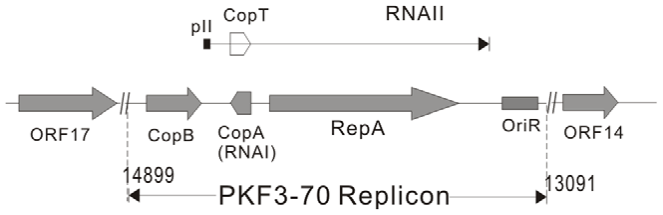
Structure of the replication system of pKF3-70. RNAII: the mRNA on which RepA was transcripted; pII: promoter for RNAII; Numbers represent the coordinates (in reference to the whole plasmid sequence).

Nucleotide Blast analysis (BlastN) revealed that a region (nt 13091-14988) in pKF3-70 is identical to the 1.9 kb replication control region of a previously reported incFII plasmid R100 (also named NR1) [8]. The replication control mechanism of FII replicon has been well studied [8], [9], [10]. Essential elements for replication in this region include a unique 149 bp replication origin (*ori*), a replication initiation protein encoded by *repA1* and its upstream negative regulators of *copA* (*RNAI*) and *copB* (*repA2*) (Fig 1) [8]. *CopA* is an antisense RNA coding gene located between *copB* and *repA1*, in the opposite strand. When it binds to its target, *copT*, it inhibits the translation of *repA1* [11]. *CopB* encodes a 9.7-kd putative protein which function as a replication negative regulator and binds near the promoter of RNAII (Fig 1).

**Figure 2:**
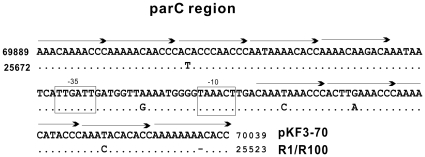
Alignment of *parC*s of pKF3-70 and R1/R100. Points in the bottom sequence (R1/R100) represent the same nucleotides as in the top sequence. “−” represents a deletion, arrows represent tandem repeats. The framed regions represent −35 and −10 boxes of the downstream parA-parB operon. Numbers at the start and end of the sequence represent the position of this region in plasmid pKF3-70.

Partition of bacterial plasmids is the process of equal distribution of replicated plasmids into daughter cells, similar to chromosomal mitosis in eukaryotes [12]. The partition system is composed of three main elements: a *cis*-acting element (a centromere-like site consisting of an array of repeat sequences), a gene encoding ATPase, and a gene encoding the centromere-binding protein [12], [13]. BlastN analysis revealed a 1.56 kb region of pKF3-70 that included a region containing 10 direct repeats followed by ORF1 and ORF2; this region had a nucleotide sequence that is almost identical (1535/1564 identities) to that of *parA* partition system in IncFII plasmids R1 and R100 [14], [15]. ORF1 and ORF2 encode putative proteins that are most similar to ParM (amino acid identity 318/320) and ParR (amino acid identity 116/117) of R100, respectively. The two loci are organized as a co-transcribed operon whose −35 and −10 promoter boxes overlap the centromere-like partition site *parC* [16]. The 149 bp *parC* contains 10 imperfect direct repeats, five of which are located upstream of the −35 box and the other five downstream of the −10 box [16] (Fig. 2). The ParR protein binds to *parC* and thereby modulate promoter activity [17]. The ParM protein exhibits ATPase activity that can be greatly enhanced by the ParR-*parC* complex [18]. The ParR-*parC* complex is also thought to mediate the pairing of two plasmids belonging to the same incompatibility group during partitioning [12].

### Conjugation System and Transfer Leading Regions

**Figure 3:**
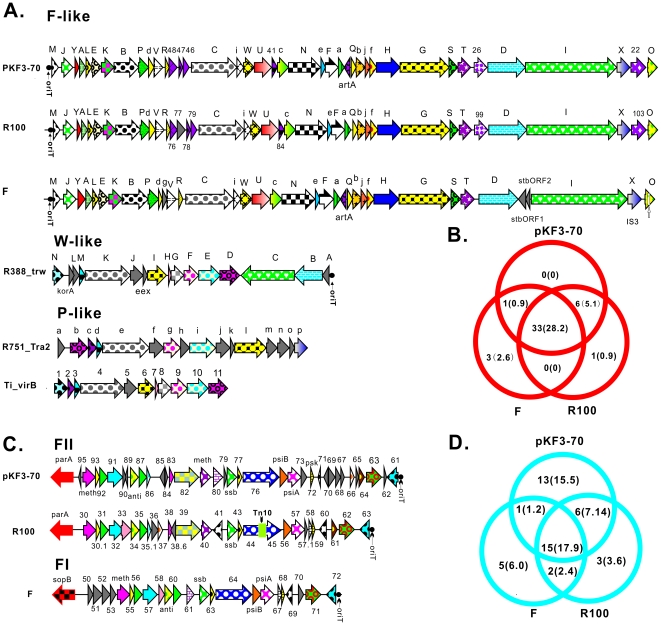
Comparative genomic analysis of conjugation and transfer leading regions of pKF3-70 and other plasmids. (A,C) show 6 conjugation regions and 3 transfer leading regions of plasmids from different incompatibility groups, respectively. Homologous genes are shown using arrows with the same color/pattern except for arrows in gray, which indicate genes without homology. The name of each gene is provided above or below. In conjugation regions of the 4 plasmids pkF3-70, R100, F and R751, *tra* genes are named in corresponding capital single letters, while *trb* genes are in the lowercase single letters. *trw* genes in R388 are marked using corresponding lowercase single letter. Numbers 1–11 in the Ti conjugation region represent corresponding VirB genes. The leading region arrows are marked using ORF or gene names. Red frame in R100 of Fig. 3C shows the transposon Tn10. (B,D) show statistics for homologous genes in conjugation regions and transfer leading regions, respectively. Numbers in brackets indicate the percentage. Except for the sequence of pKF3-70, plasmid gene information was retrieved from GenBank (accession numbers, R100: AP000342; F: NC_002483; R388: BR000038; R751: NC_001735; Ti: NC_002377.).

We have observed that pKF3-70 is able to transfer via conjugation from *K. pneumoniae* to *E. coli* and between *E. coli* strains. The 35 kb putative conjugation related region of pKF30-70 contains 40 putative ORFs (from ORF21 to ORF60) between the transfer initiation site (*oriT*) and a conjugal transfer repressor gene (*finO*). To investigate the evolution of the conjugative system in pKF3-70, we classified conjugative systems from the plasmids of incF and other incompatibility groups by their similarity to pKF3-70 (Fig. 3A). As expected, we found that the conjugation regions in three F-like plasmids were almost identical. The evolutionary distance between F-like and W-like conjugation systems was far, though slightly closer than that between F-like and P-like conjugation systems. All 40 putative genes of the pKF3-70 conjugation region had homologues in R100 or plasmid F. Interestingly, five genes (*traC*, *traG*, *traI*, *traD* and *traX*) were even conserved across incompatibility groups, implying that they might be responsible for the basic structure of the conjugation machinery. The conjugation system of F factor, a classical transmissible plasmid, has been well studied [6]. Comparative analysis of F-like conjugation systems showed that six putative ORFs (ORF22, 26, 41, 46, 47, 48 in pKF3-70) were conserved in R100 and pKF3-7 but not in F (Fig. 3A). Except for ORF22 and ORF 47, all ORFs encoded putative membrane proteins (Table 1). ORF22 had a predicted promoter located 26 bp upstream of its start site, and encoded an uncharacterized 286 aa long protein which contains an ester lipase domain implicated in lipid metabolism. The real function of this protein in the process of plasmid conjugation, however, remains ambiguous.

**Table 1:** TMHMM and SIGNALP prediction results of 6 ORFs in the conjugation region.

ORF	Length(aa)	TMHMM	SIGNALP
22	286	0	NO
26	245	1	NO
41	101	2	NO
46	115	1	YES
47	72	0	NO
48	158	1	NO

*TMHMM* column shows the numbers of trans-member domain and *SIGNALP* column indicates that whether the putative proteins contain signal peptides.

Adjacent to the conjugation region is the “transfer leading region”, which is initially transferred into the recipient bacterium during the conjugation process [19]. In F-like plasmids, the transfer leading region can be defined as the region immediately adjacent to the *oriT* and extending into the primary incompatibility determinants [6]. In pKF3-70, the transfer leading region contained putative genes related to the stability of the plasmid, such as those involved in DNA modification and post-segregation killing. Comparison of the transfer leading regions of plasmid F and its relatives, pKF3-70 and R100, indicated that they are less conserved than the conjugation regions (Fig. 3B, C and D). Except for a few genes such as *ssb*, *psiB* and the lytic transglycosylase coding gene, the genes of the leading region vary greatly across incompatible groups. The transfer leading region is about 17.5 kb long in pKF3-70 and contains 35 putative genes spanning ORF 61-95. Thirteen of these genes uniquely exist in pKF3-70 but not in R100 or F, and among them only ORF62 contains a trans-membrane domain. Six common genes exist in pKF3-70 and R100 but not in F. Four of them encode unknown proteins and the other two encode a putative adenine-specific DNA methylase (ORF81) and a putative homolog of the HD superfamily hydrolase (ORF82), respectively. One putative gene (ORF 80) is only present in pKF3-70 and F but not in R100. The transfer leading region of pKF3-70 was more similar to R100 than to the F factor. R100, however, contains a characteristic Tn10 element inserted into the *parB* gene.

### Antibiotic Resistance Gene

**Figure 4:**
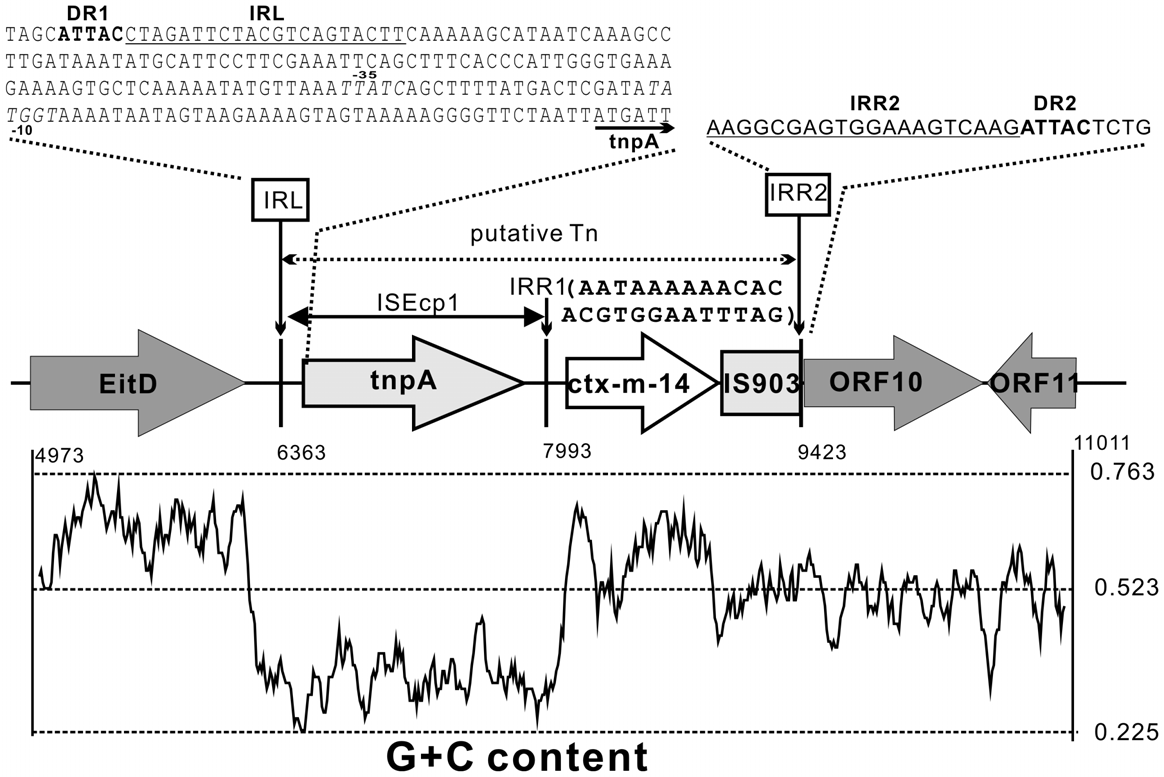
The sequence of the region containing *ctx-m-14*. DR, direct repeat; IRL, left inverted repeat; IRR, right inverted repeat; Tn, transposon. Truncated IS903 is represented with a rectangle; complete genes are represented with arrows and with gene names inside. Positions of the start points of *EitD*, IRL, IRR1, IRR2 and the end point of *YadA* are marked. These positions also serve as horizontal coordinates for the GC content map below. The map was generated using a window size of 80 bp.

Through conjugation, plasmid pKF3-70 was transferred from *K. pneunomiae* to *E. coli* J53 (AZ^R^) and the susceptibility of the strains carrying pKF3-70 to different antibiotics was examined (Table 2). The resistance phenotype of pKF3-70 is contributed to the single antibiotic gene of *ctx-m-14*. The CTX-Ms, a subfamily of extended-spectrum β-lactamases (ESBLs), have more than 40 members to date [20], and CTX-M-14 is one of the most frequently identified CTX-Ms, especially in China. The CTX-M-14 displays a high cefotaxime hydrolysis ability [21], however, it showes poor hydrolysis of ceftazidime [22] which accorded with our MIC test results (MIC of ceftazidime = 1ug/ml) (Table 2). Molecular studies showed that the structure immediately upstream of most *ctx-ms* is a specific insertion sequence ISEcp1 [20], [23], [24], suggesting that ISEcp1 might mediate the spread of CTX-M type ESBLs. In pKF3-70, the insertion sequence ISEcp1 was also found immediately upstream of the *ctx-m-14*, and a truncated insertion sequence IS903 immediately downstream. A 20 bp inverted repeat (IRR2) that matched the left inverted repeat of ISEcp1 (IRL) better than the right inverted repeat was found next to the IS903 (IRR1, Fig. 4). Moreover, there are two copies of a 5 bp direct repeat “ATTAC” precisely bordering the IRL and IRR2, thus likely the target repeats generated by insertion of a transposon. These findings imply that the region composed of ISEcp1, *ctx-m-14*, truncated IS903 and IRR2 might represent an active transposon. In other plasmids, an efficient promoter was implicated in the ISEcp1 which could promote the expression of its downstream *ctx-m*s gene [23], [25]. In pKF3-70, ISEcp1 show highly discrepant GC content when compared to nearby regions, implying different evolutionary origins. Since CTX-Ms were shown to have derived from the genus *Kluyvera ascorbata* [26], it is possible that ISEcp1 is responsible for the transmission of the resistant gene from *K. ascorbata* to an ancestor plasmid of pKF3-70 [27].

**Table 2:** Antibiotics sensitivity of *K. pneumoniae* strain J53 with and without pKF3-70.

Antibiotic	J53AZ^R^ pKF3-70+		J53AZ^R^pKF3-70−	
	MIC	result	MIC	result
ceftazidime	1	S	≤0.25	S
ceftriaxone	16	R	≤0.0625	S
cefazolin	256	R	1	S
ampicillin	≥512	R	2	S
amoxicillin	16	R	4	S

### *EitABCD* System

**Figure 5:**
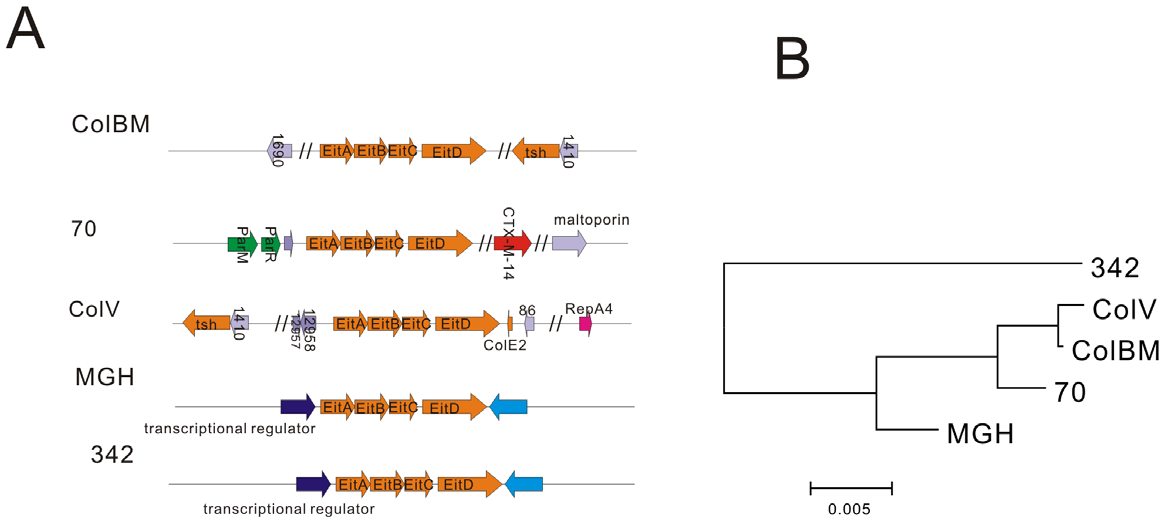
*EitABCD* system has variable context in different genome. (A) Arrows with the same color/label (except grey one) represent homologous genes. Grey Arrows represent genes of unknown functions. Numbers in the arrows represent the protein families classified by ACLAME database (A Classification of Mobile genetic Elements). Mobile elements are omitted and marked using “//”. (B) The tree was built based on the total nucleotide sequences of eitABCD. ColBM: pAPEC-O1-ColBM; ColV: pAPEC-O2-ColV; 70: pKF3-70; MGH: *K. pneumoniae* MGH; 342: *K. pneumonia* 342.

The pKF3-70 also encodes a putative iron transport system that is composed of three ORFs (ORF4-6, *eitABC*) which are predicted to be co-transcribed as an operon. The putative promoter contains ferric uptake regulator (fur) binding site, a −35 box “TTGACA” and a −10 box “TGGCATTAT”. The function and organization of these structures is annotated according to the KEGG pathway map (PATH: kpn02010). ORF4 encodes a putative periplasmic iron binding protein, ORF5 encodes a putative permease that functions as a channel through which iron passes through, and ORF6 encodes a putative ATPase. The gene directly downstream of ORF6, *eitD* (ORF7), was predicted to encode a protein that transports small solutes in response to chemiosmotic ion gradients. *eitABCD* was also found on other plasmids or bacterial chromosomes. In pAPEC-O1-ColBM and pAPEC-O2-ColV, two plasmids from avian pathogenic *E. coli*, the *eitABCD* was located in the putative virulence regions [28], [29] (Fig 5A), suggesting its probable relevance to pathogenesis. Sequence analysis showed that it has different surrounding gene contexts in the two plasmids but has the same surrounding gene contexts in two chromosomes of *K. pneumoniae* MGH and *K. pneumoniae* 342 (Fig 5A). However, the *eitABCD* sequence was more similar between the plasmids than between the chromosomes above (Fig 5B), thus it seemed *eitABCD* was transferred as an isolated genetic element and quickly spreading on the plasmids. The acquisition of putative iron transport system indicates that the genetic exchange plays an important role in adaptive evolution of plasmids.

### Comparative Genomic Analysis of pKF3-70 and Other Plasmids

**Figure 6:**
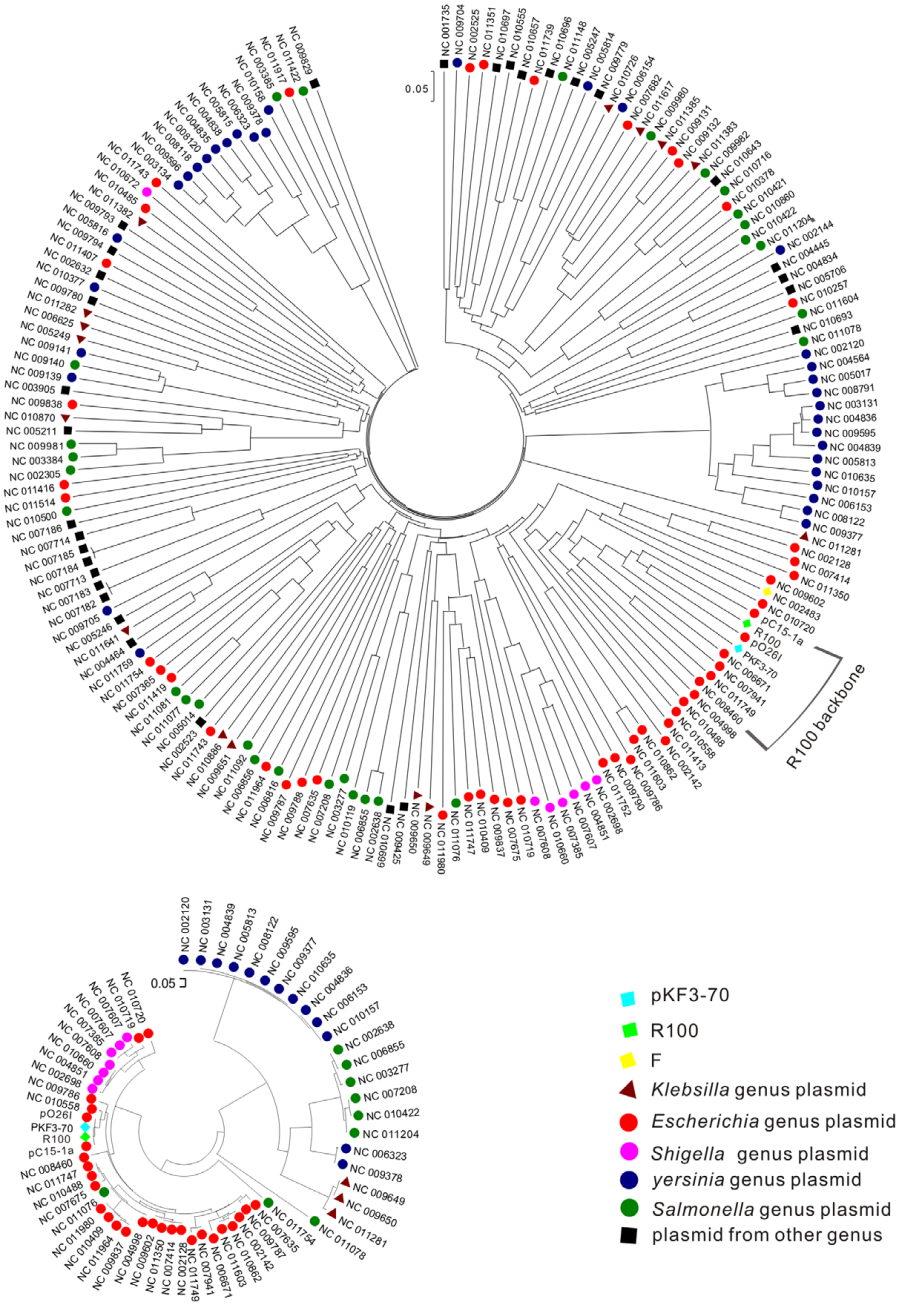
Gene-content tree and RepA tree of *Enterobacteriaceae* plasmids. Reported plasmids from Enterobacteriaceae were subjected to cluster analysis based on gene content (top) or the RepA protein sequence (bottom). Plasmids are represented by their respective NCBI accession numbers.

**Figure 7:**
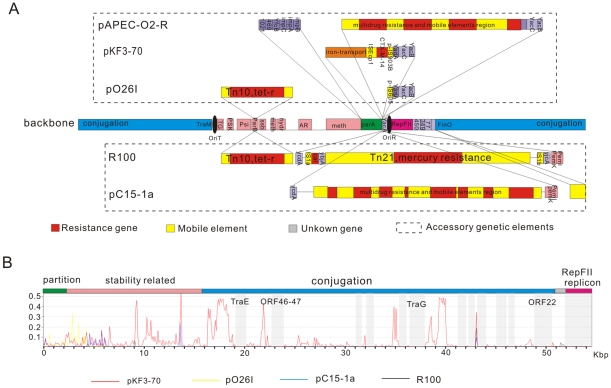
(A) R100 backbone and accessory genetic elements on different plasmids. (B) Mutation rates profile of the four plasmids along the backbone sequences. Longitudinal coordinate represents mutation rates and horizon coordinate represents position in backbone sequences. This analysis use slide window of 100 bp. Red line: pKF3-70; yellow line: pO26I; blue line: pC15-1a; black line: R100. Region with shallow grey background was absolutely identical in all four plasmids and genes in the identical regions were marked.

To shed light on the status of pKF3-70 in the evolution of *Enterobacteriaceae* plasmids, we used a gene-content method to construct the phylogeny dendrogram [30]. The traditional replication initiation protein (RepA) phylogeny dendrogram (Fig 6) are also constructed for comparison. The phylogeny relationship shown by the two dendrograms coincides in general. A general tendency could be found that plasmids from the hosts of the same genus seemed to cluster together. However, exceptions are also frequently seen, where plasmids from distant hosts cluster together while those from closely related hosts do not. For example, pKF3-70 clustered together with certain *Escherichia* or *Shigella* plasmids but far from any other plasmids from *K. pneumoniae* (Fig 6). Similarly, some *Escherichia* plasmids clustered better with pKF3-70 than most of their intra-genus plasmids. The pKF3-70 seems to be a typical *Escherichia* plasmid, though isolated from *Klebsiella* genus. Detailed genome structure analysis reveals that pKF3-70 like plasmids (Table. 3) cluster together as they contain a common 54 kb region which is an orderly organized house-keeping module encoding the genes of RepFII replicon, partition, conjugation system and stability related (Fig 7A). It is called R100-backbone and has been investigated in depth. R-100 backbone has a conserved sequence and its composition and arrangement of gene order have been genetically stable (Table. 3). The function of its conjugation system has been experimentally examined. pKF3-70 could be transferred from *K. pneumoniae* to an *E. coli* strain and maintained stably in the recipient. Previous studies also demonstrated that R100 and its derivative plasmids were conjugative plasmid and found in diverse genera [31], [32]. Conjugative transfer of cross genera or species is an important form of horizontal gene transfer (HGT) and has been observed both *in vivo* and *in vitro* [33], [34]. It might be the reason why the plasmids in similar host has the distant phylogenetic origin while closely related plasmids are dispersed in distantly related hosts.

**Table 3:** Information on selected R100-backbone plasmids.

AC_number	Plasmid	Strain	Host of strain	Isolated area	Isolated time
NC_011812	pO26I	E.coli O26	unknow	unknow	unknow
NC_006671	pAPEC-O2-R	E.coli strain APEC O2	bird	USA	unknow
NC_002134	R100	Shigella flexneri	human	Japan	1959
NC_005327	pC15-1a	E.coli clinical starin	human	Canada	2000–2002
unknow	pCTX15	E.coli	human	India	1999
FJ494913	pKF3-70	Kelbsilla.p	human	China	2006
NC_007941	pUTI89	E.coli UTI89	patient urinary tract	unknow	unknow
NC_011749	p1ESCUM	E.coli UMN026	patient urinary tract	unknow	unknow
NC_008460	pO86A1	E.coli DIJ1	unknow	unkonw	unkonw

Except the conserved backbone sequence, pKF3-70 and other R100-backbone containing plasmids vary only in certain accessory genetic elements (Fig. 7A). The region between replicon and partition module acquired more insertion of accessory genetic elements than other location. Accessory genetic elements mainly consist of highly variable mobile elements of antibiotic genes or mobile-antibiotic gene mosaic structures. They belong to a quite different evolutionary lineage and can be quickly acquired or lost through transposition or recombination. Such a changeable module likely provides plasmids with high fitness to variable environments.

PKF3-70 could be defined as a R100-backbone recombined with accessory iron transport system, *ctx-m-14* and several other genes of unknown functions. RepA tree revealed that four plasmids pKF3-70, R100, pO26I, pC15-1a have very high (even up to 100%) AA identity. A ClustalW analysis of their backbone nucleotide sequences shows that their sequence identity is not uniform among the different genome regions. This could be caused by the frequent insertion and deletion events. Nevertheless, in general, nucleotide sequences of the four backbones are highly similar. There is a 16 kb absolutely identical region including RepFII region (Fig. 7B). Considering the fact that plasmids sharing similar backbone are present in such diverse hosts (R100 from *Shigella flexneri*, pC15-1a from *E. coli* and pKF3-70 from *K. pneumoniae*), it is imaginable that in nature conjugal transfer of pKF3-70 like plasmids is frequent and fairly efficient among the bacteria of diverse genera. On the other hand, these plasmids distribute over very broad regions, R100 in Japan, pC15-1a from Canada and pKF3-70 from China, thus the spread of pKF3-70 like plasmids seems extremely efficient and fast. Apparently, public health is at stake when these plasmids carry pathogenesis associated genes, such as ESBL and iron acquisition genes. In fact, Hospital–acquired outbreak caused by bacteria harboring R-100 backbone plasmids with ESBLs are frequently reported in recent years around the world [35], [36]. As for plasmid pKF3-70, it not only carries ESBL genes, but also is the first sequenced R100-backbone plasmid isolated from *K. pneumoniae*. It indicates that R100-backbone plasmids were successful in avoiding natural barriers such as restriction systems and occupy a new ecological niche in nature. *K. pneumoniae* was one of the most common bacteria causing hospital-acquired infections and the pKF3-70 barring *K. pneumoniae* may represent a high threat to inpatients especially long-term care patients.

## Materials and Methods

### Bacterial Strains and Plasmids

The host strain *K. pneumoniae* KF3 was isolated from the laboratory of the first affiliated hospital of Wenzhou Medical College, Wenzhou, China. Species identification was conducted using a Vitek Gram-Negative Plus Identification Card (bioMerieux). The plasmid pKF3-70 is one of three plasmids isolated from *K. pneumoniae* KF3. The plasmids were extracted by alkaline lysis method [37] and pKF3-70 was further isolated by electrophoresis and purified by QIAquick gel extraction columns.

### Conjugation

Conjugation experiments were performed by liquid mating-out assay using azide-resistant *E. coli* J53 as the receptor [38]. The transconjugants were selected on tryptic soy agar plates containing 150 µg/ml of sodium azide and 64 µg/ml of ceftriaxone. The plasmid profiles of the transconjugants were examined. The transconjugant harboring single plasmid pKF3-70 was selected and the plasmid was further confirmed by the *Eco*RI digestion. Conjugation experiments were performed again using single pKF3-70 containing *E. coli* J53 strain as donor and streptomycin-resistance *E. coli* strain 600 as receptor. The transconjugants were selected on tryptic soy agar containing 200µg/ml of streptomycin and 100 µg/ml of ampicillin, then pKF3-70 in *E. coli* 600 strain was confirmed by the *Eco*RI restriction enzyme digestion.

### Shotgun Sequencing, Sequence Assembly, and Bioinformatics Analysis

Purified pKF3-70 DNA was sheared by a HydroShear DNA shearing device (volume, 200 µl; cycle number, 20; speed code, 7–8). Fragments of 1.6–3.0 kb were recovered from agarose gel electrophoresis and ligated into a SmaI digeested pUC18 vector. Clones were sequenced using an ABI 3730 automated sequencer. The sequences were assembled using the Phred/Phrap/Consed software package from the University of Washington, Seattle [39], [40], [41]. Glimmer 3.0 (default parameters) was used to predict open reading frames (ORFs) [42]. The map of GC content and GC skew is generated using CGView server [43]. Comparisons of the nucleotide sequences were made using BlastN [44]. Protein function was determined by using BlastP to search against the NCBI non-redundant protein database and the Swiss-Prot database [44]. InterproScan software was used to search against and integrated database that included PROSITE, PRINTS, Pfam, ProDom, SMART, TIGRFAMs, PIRSF and SUPERFAMILY [45]. Metabolic pathways were annotated using KEGG (http://www.genome.jp/kegg/). Multiple sequence alignments were performed using ClustalW [46]. CD-search was used to identify the conserved domains in some uncharacterized proteins [47]. Promoters were predicted using BPROM (http://linux1.softberry.com/berry.phtml) and insertion sequences were predicted using IS-finder (http://www-is.biotoul.fr/is.html). Signal peptides were predicted by SignalP [48] and trans-membrane domains by TMHMM (http://www.cbs.dtu.dk/services/TMHMM/). Gene-content tree was constructed by Blast2Network [49]. Other bioinformatics software was written using Perl and Bioperl (http://www.perl.org/) [50]. The complete sequence of pKF3-70 has been deposited in the GenBank sequence library and assigned the accession number “FJ494913”.

## Supporting Information

Figure S1 The map of GC content and GC skew of pKF3-70.
(0.61 MB TIF)

Table S1 ORFs in pKF3-70 and their annotations.
(0.21 MB DOC)
