# Supplementary figures and images for: Sequence Analysis of pKF3-70 in Klebsiella pneumoniae: Probable Origin from R100-Like Plasmid of Escherichia coli
Source: PLoS One. 2010 Jan 6;5(1):e8601. doi: 10.1371/journal.pone.0008601 (PMC2797631; doi:10.1371/journal.pone.0008601)

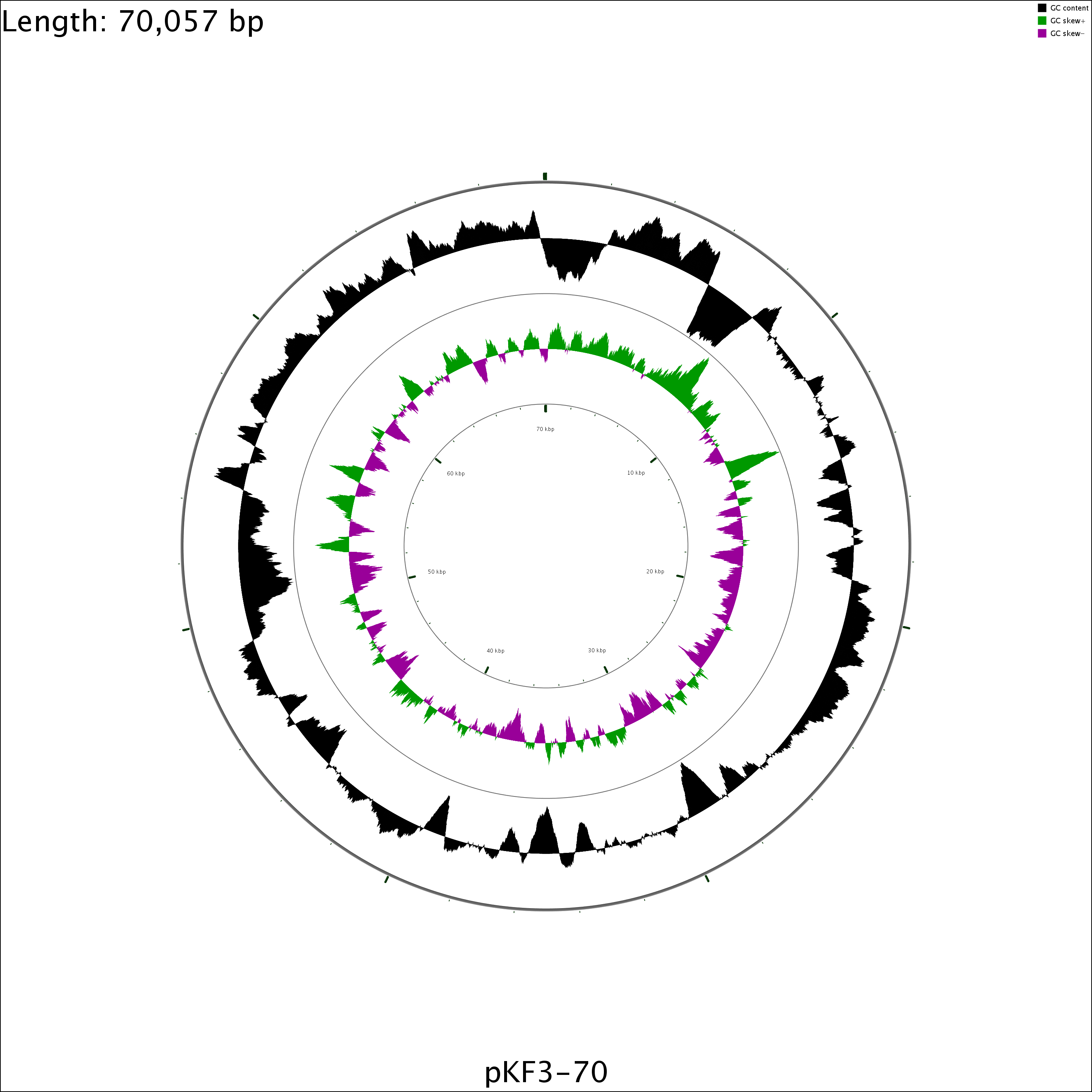

Supplement: Figure S1 — The map of GC content and GC skew of pKF3-70. (0.61 MB TIF) [file pone.0008601.s001.tif]
