# Supplementary material for: Sequence Analysis of pKF3-70 in Klebsiella pneumoniae: Probable Origin from R100-Like Plasmid of Escherichia coli
Source: PLoS One. 2010 Jan 6;5(1):e8601. doi: 10.1371/journal.pone.0008601 (PMC2797631; doi:10.1371/journal.pone.0008601)
Supplement: Table S1 — ORFs in pKF3-70 and their annotations. (0.21 MB DOC) [file pone.0008601.s002.doc]

**Table S1. ORFs in pKF3-70 and their annotations**

| **ORF** | **Location** | **Homologue** | **Best Blastp Hit** | **E-value** | **Identity (%)** | **GC**  **(%)** |
| --- | --- | --- | --- | --- | --- | --- |
| KF3.70-1 | 70056-961 | ParM | stable plasmid inheritance protein A [Plasmid R100] | 1e-180 | 99 | 39 |
| KF3.70-2 | 964-1314 | parR | stable plasmid inheritance protein B [Plasmid R100] | 3e-58 | 99 | 42 |
| KF3.70-3 | 1423-1632 | - | - | - |  | 42 |
| KF3.70-4 | 2165-3163 | periplasmic protein | EitA [Escherichia coli APEC O1] | 0 | 99 | 57 |
| KF3.70-5 | 3163-4200 | permease protein | putative iron ABC transporter, permease protein, [Klebsiella pneumoniae subsp. pneumoniae MGH 78578] | 0 | 99 | 64 |
| KF3.70-6 | 4200-4961 | ATP-binding protein | EitC [Escherichia coli] | 1e-140 | 99 | 60 |
| KF3.70-7 | 4973-6205 | permease protein | EitD [Escherichia coli] | 0 | 99 | 65 |
| KF3.70-8 | 6549-7811 | transposase | transposase [Citrobacter freundii] | 0 | 99 | 34 |
| KF3.70-9 | 8085-8936 | beta-lactamase | beta-lactamase CTX-M-14 [Escherichia coli] | 1e-156 | 100 | 61 |
| KF3.70-10 | 9469-10500 | Maltoporin | phage lambda receptor protein [Escherichia coli] | 0 | 100 | 51 |
| KF3.70-11 | 11011-10529 | putative cytoplasmic protein | YadA [Escherichia coli APEC O1] | 8e-87 | 99 | 51 |
| KF3.70-12 | 11976-11128 | DNA polymerase III, epsilon subunit and related 3’-5’ exonucleases | COG0847: DNA polymerase III, epsilon subunit and related 3’-5’ exonucleases [Escherichia coli B7A] | 1e-155 | 98 | 56 |
| KF3.70-13 | 12303-12022 | Plasmid stabilization system protein | COG3668: Plasmid stabilization system protein [Escherichia coli E110019] | 2e-47 | 98 | 52 |
| KF3.70-14 | 12569-12300 | ATPase | COG0488: ATPase components of ABC transporters with duplicated ATPase domains [Escherichia coli E110019] | 7e-43 | 97 | 50 |
| KF3.70-15 | 14340-13483 | replication initiation protein A1 | replication initiation protein A1 [Plasmid R100] | 1e-158 | 99 | 57 |
| KF3.70-16 | 14899-14645 | RepA2 | negative regulator of repA1 expression [Plasmid R100] | 9e-40 | 100 | 44 |
| KF3.70-17 | 15729-15139 | Superfamily I DNA and RNA helicases and helicase subunits | hypothetical protein R100p107 [Plasmid R100] | 1e-107 | 100 | 50 |
| KF3.70-18 | 15976-15767 | modulating protein | modulating protein [Plasmid R100] | 5e-33 | 100 | 37 |
| KF3.70-19 | 16546-16022 | thermonuclease | hypothetical protein O2ColV105 [Escherichia coli] | 1e-101 | 100 | 47 |
| KF3.70-20 | 16924-16682 | - | hypothetical protein O2R_12 [Escherichia coli] | 4e-40 | 98 | 63 |
| KF3.70-21 | 17629-17069 | FinO | Fertility inhibition protein (Conjugal transfer repressor) | 1e-101 | 100 | 55 |
| KF3.70-22 | 18592-17732 | 31.7 kDa protein | hypothetical protein R100p103 [Plasmid R100] | 1e-164 | 100 | 44 |
| KF3.70-23 | 19397-18651 | traX | F pilin acetylation protein [Escherichia coli UTI89] | 1e-141 | 100 | 58 |
| KF3.70-24 | 24687-19417 | traI | COG0507: ATP-dependent exoDNAse (exonuclease V), alpha subunit - helicase superfamily I member [Escherichia coli F11] | 0 | 99 | 60 |
| KF3.70-25 | 26858-24687 | TraD | TraD [Escherichia coli] | 0 | 98 | 53 |
| KF3.70-26 | 27646-26909 | - | orfE [Escherichia coli] | 1e-138 | 99 | 46 |
| KF3.70-27 | 28580-27849 | TraT | TraT [Escherichia coli APEC O1] | 1e-129 | 98 | 50 |
| KF3.70-28 | 29103-28594 | TraS | TraS [Escherichia coli APEC O1] | 1e-79 | 88 | 32 |
| KF3.70-29 | 31925-29100 | traG | pilus assembly protein/mating pair stabilization protein [Plasmid R100] | 0 | 93 | 51 |
| KF3.70-30 | 33295-31922 | TraH | conjugal transfer protein TraH precursor [Plasmid R100] | 0 | 100 | 52 |
| KF3.70-31 | 33674-33282 | TrbF | TrbF protein [Escherichia coli] | 3e-69 | 97 | 41 |
| KF3.70-32 | 33942-33655 | TrbJ | TrbJ protein [Escherichia coli] | 2e-46 | 100 | 44 |
| KF3.70-33 | 34477-33932 | trbB | COG0526: Thiol-disulfide isomerase and thioredoxins [Escherichia coli F11] | 1e-102 | 100 | 56 |
| KF3.70-34 | 34748-34464 | traQ | F pilin synthesis protein [Escherichia coli UTI89] | 2e-47 | 100 | 53 |
| KF3.70-35 | 34829-35164 | artA | hypothetical protein UTI89_P127[Escherichia coli UTI89] | 4e-56 | 100 | 34 |
| KF3.70-36 | 35486-35145 | trbA | inner membrane protein TrbA [Escherichia coli UTI89] | 5e-60 | 99 | 39 |
| KF3.70-37 | 36243-35500 | TraF | conjugal transfer protein TraF precursor [Plasmid R100] | 1e-142 | 100 | 52 |
| KF3.70-38 | 36496-36236 | TrbE | conjugal transfer protein TrbE [Plasmid R100] | 1e-41 | 100 | 41 |
| KF3.70-39 | 38370-36520 | TraN | TraN [Escherichia coli] | 0 | 99 | 53 |
| KF3.70-40 | 39005-38367 | TrbC | F pilus assembly protein TrbC [Escherichia coli UTI89] | 1e-116 | 99 | 54 |
| KF3.70-41 | 39319-39014 | - | hypothetical protein UTI89_P121 [Escherichia coli UTI89] | 1e-50 | 100 | 36 |
| KF3.70-42 | 40341-39349 | traU | conjugal transfer protein TraU precursor [Plasmid R100] | 0 | 99 | 55 |
| KF3.70-43 | 40970-40338 | traW | TraW [Escherichia coli APEC O1] | 1e-117 | 100 | 60 |
| KF3.70-44 | 41353-40967 | TrbI | conjugal transfer protein TrbI [Plasmid R100] | 4e-67 | 100 | 56 |
| KF3.70-45 | 43980-41350 | traC | COG3451: Type IV secretory pathway, VirB4 components [Escherichia coli F11] | 0 | 98 | 54 |
| KF3.70-46 | 44453-44106 | - | hypothetical protein R100p079 [Plasmid R100] | 2e-58 | 100 | 37 |
| KF3.70-47 | 44699-44481 | - | hypothetical protein R100p078 [Plasmid R100] | 2e-36 | 100 | 43 |
| KF3.70-48 | 45274-44798 | - | YfhA [Escherichia coli] | 1e-81 | 97 | 45 |
| KF3.70-49 | 45488-45267 | TraR | conjugal transfer protein TraR [Plasmid R100] | 3e-36 | 98 | 45 |
| KF3.70-50 | 46138-45623 | TraV | conjugal transfer protein TraV [Plasmid R100] | 1e-93 | 100 | 52 |
| KF3.70-51 | 46455-46135 | TrbD | conjugal transfer protein TrbD [Plasmid R100] | 2e-53 | 99 | 42 |
| KF3.70-52 | 47032-46442 | traP | hypothetical protein Ecol5_01003557 [Escherichia coli 53638] | 1e-106 | 96 | 50 |
| KF3.70-53 | 48449-47022 | traB | COG0419: ATPase involved in DNA repair [Escherichia coli E22] | 0 | 99 | 57 |
| KF3.70-54 | 49177-48449 | TraK | conjugal transfer protein TraK precursor [Plasmid F] | 1e-132 | 99 | 57 |
| KF3.70-55 | 49730-49164 | traE | conjugal transfer protein E [Plasmid R100] | 1e-101 | 100 | 46 |
| KF3.70-56 | 50063-49752 | traL | hypothetical protein EcolF_01003503 [Escherichia coli F11] | 1e-56 | 100 | 47 |
| KF3.70-57 | 50440-50078 | traA | F fimbriae pilin protein precursor [Escherichia coli UTI89] | 2e-54 | 95 | 46 |
| KF3.70-58 | 50701-50474 | traY | Protein traY [Plasmid R100] | 6e-25 | 61 | 40 |
| KF3.70-59 | 51468-50821 | traJ | traJ protein [Escherichia coli] | 1e-35 | 96 | 34 |
| KF3.70-60 | 52043-51660 | traM | Protein traM | 5e-64 | 99 | 40 |
| KF3.70-61 | 52364-52966 | transglycolylase | hypothetical protein O2ColV141 [Escherichia coli] | 1e-107 | 94 | 46 |
| KF3.70-62 | 53126-52992 | - | putative ORF [Escherichia coli] | 1e-17 | 100 | 53 |
| KF3.70-63 | 54083-53262 | - | hypothetical protein APECO1_O1CoBM24 [Escherichia coli APEC O1] | 1e-157 | 99 | 44 |
| KF3.70-64 | 54489-54193 | - | hypothetical protein R100p061 [Plasmid R100] | 1e-48 | 100 | 47 |
| KF3.70-65 | 54653-54513 | - | hypothetical protein O2R_63 [Escherichia coli] | 9e-16 | 97 | 58 |
| KF3.70-66 | 54967-54755 | - | hypothetical protein pU302L_028 [Salmonella typhimurium] | 1e-33 | 98 | 67 |
| KF3.70-67 | 55289-55486 | - | - | - |  | 64 |
| KF3.70-68 | 55764-55492 | - | hypothetical protein pCoo024 [Escherichia coli] | 2e-37 | 82 | 47 |
| KF3.70-69 | 56149-55793 | dopa decarboxylase | hypothetical protein KPN_pKPN4p07118 [Klebsiella pneumoniae subsp. pneumoniae MGH 78578] | 4e-30 | 57 | 47 |
| KF3.70-70 | 56175-56411 | - | hypothetical protein UTI89_P091 [Escherichia coli UTI89] | 6e-41 | 97 | 53 |
| KF3.70-71 | 56593-56724 | - | hypothetical protein pO86A1_p087 [Escherichia coli] | 2e-14 | 88 | 48 |
| KF3.70-72 | 57123-56962 | post-segregation killing protein | post-segregation killing protein [Escherichia coli] | 1e-21 | 92 | 51 |
| KF3.70-73 | 57646-57332 | Threonine dehydrogenase and related Zn-dependent dehydrogenases | COG1063: Threonine dehydrogenase and related Zn-dependent dehydrogenases [Escherichia coli B171] | 9e-56 | 99 | 57 |
| KF3.70-74 | 58362-57643 | psiA | Hypothetical protein EcolB_01004501 [Escherichia coli B171] | 1e-135 | 99 | 61 |
| KF3.70-75 | 58793-58359 | psiB | PsiB [Escherichia coli APEC O1] | 2e-77 | 100 | 57 |
| KF3.70-76 | 60806-58848 | ParB-like nuclease | hypothetical protein UTI89_P094 [Escherichia coli UTI89] | 0 | 97 | 58 |
| KF3.70-77 | 61103-60870 | putative cytoplasmic protein | hypothetical protein UTI89_P093 [Escherichia coli UTI89] | 3e-39 | 98 | 51 |
| KF3.70-78 | 61680-61159 | Single-stranded DNA-binding protein | COG0629: Single-stranded DNA-binding protein [Escherichia coli 101-1] | 6e-94 | 98 | 57 |
| KF3.70-79 | 61846-61706 | - | hypothetical protein O2R_63 [Escherichia coli] | 9e-16 | 97 | 58 |
| KF3.70-80 | 62420-61908 | - | hypothetical protein APECO1_O1CoBM13 [Escherichia coli APEC O1] | 1e-39 | 95 | 63 |
| KF3.70-81 | 63135-62572 | Adenine-specific DNA methylase | COG0827: Adenine-specific DNA methylase [Escherichia coli E110019] | 1e-101 | 97 | 59 |
| KF3.70-82 | 64542-63229 | hydrolase | COG1418: Predicted HD superfamily hydrolase [Escherichia coli 101-1] | 0 | 99 | 58 |
| KF3.70-83 | 64824-64594 | - | hypothetical protein UTI89_P086 [Escherichia coli UTI89] | 1e-36 | 100 | 58 |
| KF3.70-84 | 65057-64917 | - | hypothetical protein EcE24377A_E0030 [Escherichia coli E24377A] | 1e-16 | 91 | 45 |
| KF3.70-85 | 65083-65340 | - | hypothetical protein UTI89_P085 [Escherichia coli UTI89] | 3e-45 | 97 | 55 |
| KF3.70-86 | 66052-65861 | - | hypothetical protein R100p035.1N [Plasmid R100] | 2e-28 | 100 | 52 |
| KF3.70-87 | 66471-66049 | Collagenase and related proteases | hypothetical protein EcolE1_01003144 [Escherichia coli E110019] | 5e-71 | 97 | 57 |
| KF3.70-88 | 66943-66518 | antirestriction protein | putative antirestriction protein [Escherichia coli] | 1e-80 | 100 | 54 |
| KF3.70-89 | 67100-67240 | - | hypothetical protein EcolB_01004617 [Escherichia coli B171] | 1e-19 | 97 | 64 |
| KF3.70-90 | 67359-67192 | - | unknown; orf44 [Escherichia coli] | 5e-08 | 56 | 61 |
| KF3.70-91 | 68186-67359 | truncted DNA methyltransferase | hypothetical protein NR1_p033 [Escherichia coli] | 1e-148 | 96 | 60 |
| KF3.70-92 | 68620-68186 | Transcription-repair coupling factor (superfamily II helicase) | hypothetical protein EcolB_01004390 [Escherichia coli B171] | 3e-73 | 95 | 58 |
| KF3.70-93 | 68855-68634 | - | hypothetical protein ColIb-P9_p035 [Plasmid ColIb-P9] | 3e-34 | 98 | 55 |
| KF3.70-94 | 69539-68856 | DNA methylase family protein | DNA methylase family protein [Escherichia coli E24377A] | 1e-128 | 96 | 57 |
| KF3.70-95 | 69646-69771 | - | hypothetical protein SC131 [Salmonella enterica subsp. enterica serovar Choleraesuis str. SC-B67] | 2e-12 | 87 | 61 |
